# Researches into the Causes, Nature, and Treatment of the Diseases of Warm Climates

**Published:** 1829-04-01

**Authors:** 


### Medico-Chirurgical Review,

### No. XX.

JANUARY 1, to APRIL 1, 1829,

I.

### Researches into the Causes, Nature, and Treatment of the Diseases of Warm Climates, By *James Annesley*, Esq. Two Volumes, 4to. 1827—8.

[Art. III.—Chronic Hepatitis, and Organic Diseases of the Liver.]

Of all the diseases to which Europeans are particularly prone in Tropical climates, chronic hepatitis is the most interesting to the home department of medicine in the British Isles. When the liver has taken on this form of disease, the invalid generally returns to Europe, where he continues to be, at once, a plague and a profit to every practitioner within his reach during the remainder of his life. As for Cheltenham and Leamington, their very streets are paved with the pagodas that have been purchased in the East at the expense of health in the West! But this is not all. It is suspected—nay, it is truly affirmed, that liver disease is transmitted to the offspring of the Anglo-Indian, with as much certainty and facility as pulmonary tubercles, gout, or mania. And why should not the hereditary disposition be transmitted in the one case as well as in the others? No reason to the contrary can be assigned, while observation is in favour of the affirmative side of the question. Considering the prodigious influx of oriental and occidental invalids annually into this country, most of them labouring under chronic disease of the liver, the evil would be most alarming, did not the nature of our cold and gloomy climate check the hereditary propagation of hepatic diseases, in the same way that the bright skies and fervid atmospheres of the Eastern and Western Indies retard the progress, or annul the propensity to pulmonary phthisis. But still the class of maladies under consideration is quite extensive enough to render its investigation a matter of very great importance among all classes of the profession in this country.

Few have had more ample opportunities of seeing the disease under consideration, on a large scale, than the author of the great work now before us; and, therefore, we propose to give a very full account of this portion of the publication, interspersed with original and collected information, wherever we see an opportunity for its introduction.

Chronic inflammation of the liver may occur as a primary disease, or take place as the sequel of acute disease.

"When chronic inflammation takes place primarily, it generally is seated in the internal texture of the organ, and often gives rise to but few local symptoms, and but little constitutional disturbance. But chronic is a term which conveys with it no precise idea, and merely signifies a slow state of inflammatory disorder, presenting every grade, from that state of disease which may be considered as only slightly deviating from the healthy action, and which may continue for a great length of time, giving rise to various organic changes, to that which runs its course rapidly, and terminates, either one way or another, in a very few weeks. When this form of inflammation remains after the more acute phenomena have been subdued, it has generally its seat in the substance of the liver, but not uniformly: it may be seated in the surfaces; for the active inflammation, which has been followed by the effusion of coagulable lymph upon the surface of the organ, and the formation of adhesions between it and adjoining parts, may be, to a certain extent, rekindled, after it has been altogether or nearly extinguished, and the vascular action reinduced may assume a slow and sub-acute form. It should also be recollected, that, although chronic inflammations of the liver may follow upon acute attacks, the latter may also supervene to the former, and actually do so on many occasions, particularly when the patients have been exposed to energetic exciting causes, or to an injudicious regimen and treatment. This should always be kept in mind during the treatment of both acute and chronic forms of inflammation of the liver; for it should be an object of importance with the practitioner to prevent active inflammation from degenerating into chronic, and the chronic from being converted into active disease." 470.

Chronic inflammation of the organ in question generally commences with, and must necessarily be accompanied by, deranged function of the same. The biliary secretion is either depraved in quality, or its flow into the duodenum obstructed. It is seldom in due quantity, though occasionally it seems overabundant—a phenomenon which our author thinks is owing to a previous impediment to its free egress from the liver. The diminution of the biliary secretion, upon the whole, predominates, as may be inferred from the appearance of the fæces, and the defective state of the digestive and assimilative functions.

As chronic disease of the liver varies, *in grade*, from active inflammation, down to the most trifling deviation from healthy function, we may readily conceive the great variety of symptoms which must present themselves, and the utter impossibility of delineating them all. Add to this, the complication of stomach disorder, intestinal irritation or torpor, nervous derangement, &c. which almost invariably accompanies the hepatic affection!

"The loss of flesh; the dyspeptic symptoms, particularly the slow and painful digestion, accompanied with acid and acrid eructations, flatulency, nausea, and sometimes vomiting, torpid state of the bowels, or dark-coloured, offensive, slimy, greenish-coloured, tenacious, or watery and muddy motions; the frequent calls to stool, and the scanty and morbid state of the evacuations; the dark-coloured and disordered condition of the urine; the distension and oppression at the epigastrium and right hypochondrium; the occasional aching pain and weight in these situations; the uneasiness and pain about the right shoulder or shoulder-blade; the slight acceleration of the pulse towards evening, with an irritable beat, and considerable heat and restlessness through the night; the burning heat of the palms of the hands and soles of the feet in the evening, and chilliness in the morning; the white, foul, and excited tongue; the bitter or disagreeable taste of the mouth; the hardened state of the gums; the sallow and tallowy appearance of the countenance, and either yellow or pearly-white colour of the eye; the sickly and leuco-phlegmatic character of the body generally; and the elevation of the shoulders,—are the principal symptoms by which we are guided in determining the existence of chronic inflammation of the internal structure of the liver." 472.

It is when any of the surfaces of the organ are engaged in the inflammatory process, that pain is more complained of. When the *convex* surface is implicated, the pain is more referred to the chest—when the *concave*, to the stomach and bowels. In every case, there should be instituted a careful examination of the parts by the hand and the eye. The manual examination should be conducted with gentleness, and no unnecessary pain excited, lest chronic inflammation may be kindled up into an acute form.

The terminations of chronic hepatitis are various. The greater number of them may be looked upon as advanced stages of the inflammatory state—others merely as organic changes to which this state invariably leads in particular habits and constitutions. The chief organic changes met with in the East, consequent upon chronic inflammation of the liver, are the following :—

"Collections of matter may form in the substance of the organ consequent upon chronic inflammatory action, as well as from the more active state of disease. When the purulent matter is collected into one large abscess, it generally nearly approaches the appearance of abscess consequent upon active inflammation, and will receive attention when the subject of abscess comes specifically under consideration. Not infrequently, however, very minute abscesses are scattered through the substance of the liver, both with and without the appearance of a distinct cyst, the matter collected being of a firm or cheesy consistence, and yellowish white colour. Sometimes this consistent kind of matter does not fill completely the cavity containing it: it seems as if the watery portions of the matter had been removed by absorption, and thus the more consistent part fills imperfectly the cavities in which it is contained. The substance of the organ intervening between the purulent deposits is sometimes more vascular than usual, and of a brick-red colour: and at other times not materially changed from the healthy colour and consistence.

"The liver, in many instances of long-continued and slight inflammatory action, becomes much enlarged, particularly its right lobe. This appears to arise from the deposit of lymph in the interstices of the structure, which deposit becomes dense, and closely resembles an organized substance, most probably from the absorption of its watery portions. The enlargement is often accompanied also with deposits of purulent matter in various parts of the organ, with a friable state of its texture, and a dark-coloured and congested condition of both its internal structure and surfaces: the latter are generally much darker than natural, and often variegated with lighter streaks and small spots.

"When the deposition of lymph in the structure of the liver is attended with greater density of its orgânization, either partially or generally, the change has been ascribed to a specific organic change; and a true scirrhous condition of the organ has been considered as the result. This state seems to us to be merely the consequence of very slow inflammatory action, with a deposit of organized matter, and an increased consistence of the reticulated or cellular parenchyma of the viscus, and frequently with an effusion of lymph in the granulated tissue composing the greater portion of its internal structure. It seems to us also, that the consistence of the organ met with in cases of chronic disease characterised by enlargement, is more the consequence of the activity of the inflammatory action from which it proceeds, and the habit and constitution of the patient, than any other cause; the organ being more friable and congested, the more acute the previously existing disease,—and more firm and more closely resembling a true scirrhous and semi-cartilaginous state, the more chronic or slow the inflammatory action which had existed.

"Tubercles of various kinds,—some apparently encysted, others without any evident cyst or distinct envelop, and, when divided, presenting either a concentric or radiated texture, varying in consistence from a gristly or cartilaginous state to one of semi-fluidity, occasionally filling completely the cavities in which they are contained, particularly when they approach a state of fluidity, and at other times, when their consistence is greatest, leaving vacuities between their circumference and the parts of the liver surrounding them—are often severally detected in examinations of the more chronic forms of hepatic inflammations. In many cases, the substance of the liver containing these tubercular formations presents little or no evidence of much inflammatory action having existed, at least recently, in the organ. The tuberculated liver is often also enlarged, and occasionally it is much firmer in its texture than usual. When signs of co-existent inflammation of the internal structure are present, there is frequently also greater friability; but this is not uniformly the case. Sometimes the substance of the viscus presents a gristly or cartilaginous appearance, and is lacerated with greater difficulty than usual. Such appearances are chiefly remarked in the most chronic cases.

"In these cases also, more particularly in those addicted to the use of spirituous and intoxicating liquors, the substance of the liver is obscurely tuberculated, of a cheesy consistence and texture, and of a deep nankeen-like colour: it is generally, at the same time, more or less enlarged. In many chronic cases of diseased liver, arising from the above cause, we have found the internal structure of the organ of a parboiled and scabrous appearance, drier and more spongy, than natural, and, when divided by a scalpel, or torn asunder, presenting a more or less pale colour, and great inequality of consistence, small rough eminences being surrounded by soft, grayish, and spongy matter. In some of these cases, the substance of the viscus is of a grayish-brown colour. Conjoined with this condition, the size of the liver is often diminished, its vessels nearly without blood, the hepatic ducts devoid of bile, and the gall-bladder either empty or containing a small quantity of a pale, straw-coloured, watery fluid, scarcely resembling bile. The state of the hepatic vessels, biliary ducts, and gall-bladder, is often also conjoined with scirrhous enlargements, tuberculous disease, with atrophy, and with many of the other very chronic states of the liver now described." 476.

When enlarged and tuberculated, the liver may generally be felt projecting from under the margins of the ribs, especially on the right side; "but such forms of organic change are less frequently observed in India than in Europe." The functions of the organ are always greatly disordered, according to our author's experience, in these cases of tuberculation, and other organic changes. The biliary secretion is diminished, and its colour unhealthy—the digestive and assimilating functions are impaired—the body wastes—there is drowsiness and pain over the eyes—bad taste in the mouth—a tallowy or unhealthy appearance of countenance—irregular condition of the bowels, with pale, morbid, and offensive stools—high-coloured urine—slight evening fever.

In many cases of very chronic hepatitis, the liver becomes *atrophied.* It is extremely difficult to pronounce upon such state, though it may be suspected by the exceeding torpor of the biliary functions, the deficiency of bile, "and the sunk state of the epigastrium and margin of the false ribs, when the patient is in the reclining position." These symptoms, however, cannot be relied upon.

The post-mortem appearances met with in cases of chronic inflammation of the liver are, abscess, and small collections of matter—tubercles—enlargement of the organ and softening of its texture—induration and scirrhous tumours—enlargement with friability, or cartilaginous hardening of structure—a rough, pale, and parboiled-like appearance—a cheesy and tuberculated state of its structure—a spongy and less vascular condition of its internal texture—atrophy, with or without the vestiges of cicatrices—various colorations of its surface or substance—adhesions.

The above organic changes are frequently met with in fatal cases of dysentery—especially of what is called hepatic dysentery, to be hereafter described; and in bilious remittent fevers, or obstinate intermittents. Indeed the said changes are more frequently observed as complications than as simple diseases of the biliary apparatus.

In addition to the foregoing catalogue, there are other diseases affecting the gall-ducts—as collections of viscid and inspissated bile—or, biliary calculi. The latter, however, are comparatively rare in the ducts, though often found in the gall-bladder. These obstructions and concretions produce the same phenomena in hot, as in cold, climates. Inflammation of the pancreas and duodenum often extends to the concave surface of the liver, producing jaundice, and the other phenomena of hepatitis, with additional pain in the region of the duodenum, proceeding from beneath the right scapula to the right hypochondrium, with a sense of dragging or drawing together of the parts in the neighbourhood. In such states, the opening of the ductus communis will be partially or totally obstructed, and jaundice will be the consequence. It is hardly necessary to say, that jaundice can only be viewed as a symptom of some disease of the liver, or of some mechanical obstruction to the issue of bile from that organ into the intestines. It is unnecessary to say any thing of the causes of chronic hepatitis, after what has been said of the etiology of acute hepatic inflammation, in a former article. Functional derangements, however, and a vitiated condition of the bile itself, by keeping up constant irritation in the bowels, and in the system generally, are no uncommon causes of actual inflammation, acute or chronic, in the biliary apparatus. Sub-acute inflammation of the liver, if taken in time, and judiciously treated, generally ends favourably—and when the termination is fatal, it is usually by the induction of organic disease in the liver or large bowels. We shall introduce the following case in illustration.

"CASE LXIII.—*Chronic Inflammation of the Liver, with Congestion and Secretion of Morbid Bile; Inflammation of the small and large Intestines supervening, and terminating the Life of the Patient.—Examination post Mortem.* (*See Plate X.*)

"WILLIAM MAC LAUREN, aged 35, was admitted while encamped at Kurnool, 19th October. Is a debilitated man, from long residence in India, and from drinking the intoxicating liquors of the country. Had, a few months since, an attack of chronic hepatitis, with disorder of the bowels, from which he apparently recovered. Has been again complaining for some time of hepatic symptoms, and has now purging; passes blood by stool: great sickness at stomach, and bitter taste in his mouth; tongue foul.—Habeat haust. emet.

"*Vespere.*—He vomited a lumbricus; considerable debility.—℞ Aquæ ammon. ♏xx.; spirit. lavand. compos. ʒij.; aquæ puræ, ℥jss. Ft. haust. stat. sumend. Habeat hydrarg. submur. gr. xij. h. s.

"20th.—Pulse 80, and good; tongue foul and yellow; great thirst; stools bloody, mixed with mucus, and some fæces.—Habeat ol. ricini, ℥ij. Injiciatur enema purg. statim. Habeat mist. salin. ℥ij. secundâ quâque horâ.

"*Vespere.*—Pulse 90, more languid; tongue foul; thirst urgent; stools the same as in the morning.—Cont. mist. salin. Adhibeat scrob. cord. emplast. lyttæ. ℞ Hydr. submur. gr. xij.; pulv. antim. gr. iij.; opii, gr. ij.; syr. q. s. Ft. pilul. h. s. Injiciatur enema emolliens.

"21st.—Pulse the same as at last visit; tongue still foul; stools bloody.—℞ Ol. ricini, ℥jss.; aquæ. menth. pip. ℥ij. Ft. haust. stat. sum. Injiciatur enema. cum ipecac.

"*Vespere.*—Pulse 108; skin very moist; tongue foul and dry; stools watery, bloody, and mucous; great flatulence; no pain of the belly when pressed.—℞ Hydr. submur. ℈j.; opii, gr. ij.; syr. q. s. Ft. pilul. h. s. s. Injiciatur enema emolliens.

"22d.—Pulse 98, much weaker; skin agreeably moist; tongue still foul; stools liquid and bloody; some griping pain in the night; great prostration of strength.

—Injiciatur enema emolliens. ℞ Acid. nitros. ʒij.; tinct. opii, ʒiij.; aquæ puræ, ℔ij. Fiat potus, cujus cyathum horis singulis sumat.

"*Vespere.*—Pulse frequent, and very feeble; tongue furred; thirst urgent; stools unaltered; tenesmus less severe; no pain.—Contin. pot. acid. heri præscriptus. Injiciatur enema, cum ipecac. Habeat haust. anodyn. h. s. Bibat infus. tamarind.

"23d.—He has been very uneasy during the night, and has vomited a great deal; pulse imperceptible; skin cold, and exhaling an unpleasant odour; stools watery and bloody; no pain; he appears to be moribund.—℞ Tinct. opii, ♏xl.; spirit. æther. sulphur., aquæ ammon. utriusque, ♏xx.; aquæ puræ, ℥ij. Ft. haust. stat. sumend.

"*Vespere.*—No improvement of the symptoms.—Cont. med.

"24th.—The body exhales a strong cadaverous smell, and all the symptoms are worse.—Ponantur juxta lectum aceti acetabula vaporantis. He died at one o'clock.

"*Examination, two hours after Death.*—The liver was much enlarged, and of a blackish brown colour externally, and its right lobe rose high into the right thorax, and was of a spheroid form. Upon being divided, its vessels were greatly congested with blood, and its ducts with thick, dark-coloured, viscid bile. The internal structure of the liver was more than usually vascular, of a dark brick colour, and more condensed and more friable in its texture than natural. The gall-bladder contained some green-coloured bile. The ducts were unobstructed. The small and large intestines were inflamed throughout, and so much softened in their texture, and so easily lacerated, that the ilium and colon tore upon removing them for inspection. The mucous coat was detached from the subjacent texture in many places of the colon, and in others it was sphacelated. The other organs presented no marked derangement.

"*Remarks.*—We did not see this case until symptoms of gangrene of the bowels had supervened; otherwise, notwithstanding the circumstance of the worn-out system of the patient, and his inordinate addiction to spirits, depletions would have been practised. The time for their employment soon passed off; and after the 21st, his case admitted of no hopes from any treatment. The liver presented an appearance of disease very frequently met with as a consequence of chronic inflammations of the organ, (see Plate X.); and on that account the case is inserted in this place." 483.

The plate shews the morbid appearances above-mentioned in a very striking manner.

### ABSCESS OF THE LIVER.

This termination is very common in India, if active means are not early employed in the inflammatory stage—or, if the disease creeps on insidiously, and eludes attention. The sanguine and scrofulous habits are particularly prone to suppuration of the liver.

"It may with great justice be dreaded by the practitioner, when he finds, upon examination, considerable tumefaction of the organ accompanying the early stages of the disease;—and that it frequently supervenes to the insidious inflammation of the substance of the liver, which often accompanies, if it does not actually occasion, a particular variety of dysentery, and which, although not generally manifested by acute symptoms referable to the region of this organ, is not the less active as respects its progress and termination. Indeed, in many instances, the practitioner of extensive practice in India, will find, when the early stage of inflammation of the liver is accompanied with much fever, a heavy aching pain, and great tumefaction in the region of the organ, that it is very difficult to prevent the supervention of suppuration even by the most prompt and copious depletions, and by the most active employment of mercurial remedies. It frequently happens, also, that considerable enlargement of the liver is observed as a sequela of active disease of the viscus, even although much decision may have been evinced in the treatment, and the most urgent symptoms have been subdued. But, in such cases, enlargement of the organ is the result of some degree of effusion of lymph in the interstices of the inflamed tissue, and denotes a similar state of parts to that marking the previous existence of inflammatory action in more superficial and more tangible glands." 518.

Some cases of hepatic abscess are given in detail by our author, as illustrations; and then he proceeds:—

"When acute attacks of hepatitis are not subdued by sufficiently decisive treatment in their early stages, they run rapidly into abscess. This consequence of the disease is chiefly to be dreaded when considerable enlargement of the viscus is found upon examination. If abscess actually be formed, and is seated in the convex part of the right lobe, the enlargement is evident over the whole hypochondriac region, the liver extending considerably below the ribs towards the umbilicus, and sometimes across the epigastrium to the left side. When the abscess is likely to point below the ribs, there are generally great tumefaction and increased heat of the surface of the part and its vicinity: frequently there is found a distinct enlargement, particularly in the more advanced progress of the abscess, immediately under the margin of the right ribs. If, however, abscess form on the superior surface of the liver, and point upon the diaphragm, although the enlargement of the organ will be very perceptible, yet there will seldom be felt any great increase of temperature on the surface of the hypochondrium. The abscess may point between the ribs; in this case, a bulging of the false ribs will be observed, and more than usual fulness of the intercostal spaces, and increased heat in this situation, with considerable enlargement, the liver being felt below the right hypochondriac region, in the epigastrium, and sometimes in the left hypochondrium. This enlargement may exist for a considerable time before matter forms; but in this case there will be no distinct tumour nor increase of heat: when the abscess has advanced considerably to maturity, the undefined enlargement and tumefaction become even diminished, and distinct tumour is more observable, according to the situation of the abscess and the direction which it may take.

"When the abscess is completely formed, and is seated in the superior and posterior part of the liver, the enlargement and tumefaction felt beneath the ribs, previous to, and during the formation of matter, become considerably diminished; but if it be in the inferior and anterior part of the organ, the enlargement becomes more and more reduced and circumscribed, until it assumes the character of a distinct tumour; and the pain, which was often considerable during the period of general enlargement or tumefaction, either altogether ceases, or is now but little felt. For further observations characterising external pointing of abscess of the liver, we must refer our readers to the section on treatment of abscess, and on the operation for abscess when it points externally." 527.

The formation of hepatic abscess is not always revealed by unequivocal symptoms, particularly when it supervenes on chronic inflammation, and complicated with dysentery. In such cases the matter may form without a single symptom usually denoting abscess of an internal organ. The disease is so complicated with aguish feelings, that rigors or horripilations are fallacious phenomena. In general, however, minute inquiry will detect the existence of slight shudderings or formications, in the absence of unequivocal rigors.

"Sometimes an internal sense of throbbing and fluttering has been felt in the region of the liver, and has been followed by a broad, soft pulse, and night perspirations. The supervention of night perspirations, with a clamminess of the skin of the extremities, is one of the most certain signs of the formation of internal abscess which we possess; but even this ought not to be relied upon alone, but should be viewed always in connexion with the other symptoms characterising the case. The next in importance are frequent cold sweats, but these are chiefly met with in the advanced stage of abscess. Frequent fainting sensations are deserving of considerable reliance on the part of the practitioner. There are also generally much anxiety and oppression at the precordia, and restlessness. If, during the treatment of hepatitis, we find it a matter of difficulty to affect the system with mercury, vascular depletions having been previously practised with the requisite decision, we may then dread the existence of abscess. Whether or no the mercurial remedies employed may act in such cases, owing to peculiarities of constitution or diathesis, in producing and accelerating the suppurative process, is a question not readily admitting of decision; but there can be no doubt that the system will not be brought under the full operation of mercury, or that ptyalism will not follow upon the most energetic employment of this substance, when abscess exists, although a slight tenderness of the gums will be produced by it. This circumstance has been proved to us on very many occasions.

"When abscess is formed, the tongue is seldom or ever of a natural appearance. At first it is sometimes white, and the papillæ raised or excited: it afterwards becomes of a dusky, brick-coloured redness, or what may be called a beef-steak tongue. At other times it is dry, coated, and of a brown tinge. In the more chronic cases, it is often smooth, chapped, lobulated, and apparently deprived of its papillæ. When great mischief is going on in the liver, without any acute symptoms, the tongue is often an excellent guide, and more to be depended upon than the pulse. In many of the less acute or chronic cases of abscess, the tongue has a peculiar white appearance, with the papillæ raised or excited: it is somewhat dry, but without any coating. This is what we have called an excited tongue, because we have considered it a sign of great vascular excitement going forward in internal structures; and we have often ordered depletions from this symptom alone, the tongue becoming natural as soon as a full depletion was performed. Hence we have considered that, when this state of tongue is observed, depletions may be directed more safely than upon the indication of any other symptom. Care should, however, be had not to confound this appearance with a white and moist condition of the tongue, or with a white, yellow, or brown crusted state of this part. The pulse, at the commencement of the formation of matter, is generally soft and full, is subject to acceleration in the evening, and, as the organic change advances, becomes more irritable, quick, and contracted. The stools are always much disordered through the progress of abscess of the liver: they are generally more or less frequent, are scanty, and usually consist of a greenish, watery fluid, with a greenish froth, or a green, slimy scum, floating on their surface. Frequently there are also straining and tenesmus; and some blood, with mucus, is occasionally voided. The calls to stool are also, in many cases, most frequent during the night. In hepatic disease, terminating in abscess, and complicated with dysentery, both the small and large intestines become diseased,—first functionally, and afterwards organically; and the patient generally dies of the organic change produced chiefly in the large intestines, frequently before the abscess makes its way, either externally or into any other organ. In many cases of hepatitis, complicated with dysentery, more particularly when the hepatitis presents a chronic character, the termination of the inflammation in abscess is accelerated, if it be not altogether produced, by the sudden arrest of the dysenteric disease. In many other cases, as we shall have to shew more fully when hepatic dysentery comes before us, the hepatic disease is not apparent until the dysenteric symptoms are subdued; but although the disorder of the liver was not evident, or did not excite notice, while the bowel disease was urgent, we are not on that account to infer that it did not then exist. On the contrary, we believe that in most of the instances of this description the liver was the original seat of mischief, which only became more severe and more apparent when the consecutive disorder was abated." 530.

When the abscess is seated posteriorly in the liver, and presses on the diaphragm, anxiety and præcordial oppression are urgent, with occasional dyspnœa or hiccup—when pointing towards the stomach, flatulence and vomiting are seldom absent. The easiest position is usually on the back—sometimes in the sitting posture. Pain is a very uncertain symptom. A pricking sensation is commonly felt where the abscess is pointing. The countenance is indicative of disease—it is sallow, sunk, the eye of a pearly hue—tongue white and excited—spirits depressed, sometimes even to melancholy—progressive emaciation—quickness of pulse in the evening—bowels either relaxed or costive—motions morbid, "and always deficient of healthy bile," resembling soft clay or putty, with a peculiar fetor. In such cases we may infer, that the mischief is not yet very extensive, but that it will be not the less serious in the end.

"When, on the other hand, the symptoms are more acute, when the constitutional derangements are great, the tongue dry and smooth, the fever very considerable, and the functions of the alimentary canal much disturbed, and signs of dysentery present, then immediate danger is to be apprehended." 531.

Abscess of the liver may wear out the constitution before it breaks externally or internally—generally through the medium of a bowel-complaint. The appearances on dissection are very various, not only as regards the situation and extent of the abscess, but the colour, consistence, &c. of the purulent formation. The state of the surrounding parenchymatous structure is also very various. These varieties we need not dwell upon. Neither can we afford space for any of the numerous cases which are detailed by the author.

### TREATMENT OF CHRONIC HEPATIC INFLAMMATION.

In our second analytical article on Mr. Annesley's work (No. 18. p. 344.) we took up the treatment of *acute* hepatitis, and, therefore, need not again go over that ground.

Mr. Annesley observes that, the chronic forms of hepatitis differ only from the more acute, in the duration of the disorder, "and in the texture of the organ, more generally the seat of the inflammatory action." In fact, he considers the division into acute and chronic as entirely arbitrary, and only to be adhered to as far as respects the "duration of disease." The therapeutical agents are ranged under the following heads.

I. *Vascular Depletion.* Whether chronic hepatitis has resulted from the acute form, or been primary in itself, more or less vascular depletion will be necessary—chiefly local. The extent must be left to the judgment of the practitioner. The vigour of the patient's constitution, the fulness or pain in the side, the febrile action in the system, and the length of residence in a hot climate, will afford the necessary indications. There are few cases, however, where repeated, though moderate leechings will not be beneficial. After each leeching Mr. A. recommends a poultice to the side, and an aperient, consisting of calomel at night, and black draught in the morning. When the morbid action of the liver is lessened by these means, and the morbid secretions carried off, the calomel may be changed for a milder preparation, as blue-pill, or the hydrargyrum cum creta, with saline and antimonial aperients. Bowel-complaints are very common attendants on chronic hepatitis.

"When such is the case, enemata, either of a purging or of an emollient nature, should be administered, and the pulvis ipecacuanhæ comp. given in combination with the blue-pill at bed-time, and be followed by a dose of castor oil in the morning. In many of these cases, much advantage will be derived from the use of a flannel bandage kept constantly applied round the abdomen; and the local depletions which have been practised may be followed by blisters on the epigastric or hypochondriac regions, and these by the nitro-muriatic wash, until a healthy state of the secretions be brought about." 627.

II. *Nitro-muriatic Solution.* After the removal of acute symptoms by the means already pointed out, Mr. Annesley strongly advocates the use of this remedy, which English practitioners have strangely neglected—or rather rejected, without cause. We are in the habit of prescribing it frequently—and we are as certain of its effects on the system as we are of jalap or calomel. The following is Mr. A.'s formula for the solution.

"The nitro-muriatic solution, lotion, or bath, may be made in the following manner:—Into a common quart bottle put about eight ounces of pure water, to which add four ounces of the nitric acid, and four of the muriatic acid, of the strength of the London Pharmacopœia. The '*Nitro-muriatic Solution*' is thus formed, and the bottle containing it ought to be labelled accordingly. If it be intended to use this solution in the form of a bath, from 2 ounces of it to 5, according to the strength of the patient, may be mixed with from two and a half to three gallons of warm water, of a temperature nearly approaching that of the blood, in a high and narrow vessel, and the feet and legs kept immersed in it for about twenty minutes or half an hour, every night before retiring to rest. If the bath does not occasion a pricking or itching sensation in the parts immersed, after twenty minutes have elapsed, the next bath should be increased in strength. Although we have frequently employed this bath, and generally with advantage, we prefer, in many respects, the practice of sponging the trunk of the body, particularly the abdomen, with the nitro-muriatic wash.

"When the nitro-muriatic solution is to be employed in the form of a wash, from two to three drachms of the *Solution*, prepared as just stated, should be added to a pint of warm water, and the trunk of the body, insides of the thighs, &c. assiduously sponged with it, by means of a large sponge, for about a quarter of an hour daily, or, occasionally, night and morning. We have found great advantage from employing this solution also in the form of poultice, in torpor of the liver and in chronic affections of the organ, attended with enlargement and a deficient and morbid state of the biliary secretion. Occasionally much benefit will arise from employing this wash in the form of fomentation; the water having been made as hot as 130° or 140° of Fahrenheit, when the acid solution is added. When this is practised, the flannels soaked with the wash should be applied for about an hour or two every night. It may be employed, also, with advantage by keeping cloths wet with the solution over the hypochondria and abdomen, and placing over them warm poultices; both the moistened cloths and the poultices being renewed from time to time." 628.

Mr. A. assures us, and we believe him, that he has seen the most decided advantages result from this remedy in chronic hepatitis, "and, indeed, in all functional disorders of the liver." In the more chronic forms of disease of the liver, he observes, particularly where the structure is enlarged, and the biliary and intestinal secretions vitiated, "I consider it one of the most valuable remedies we possess." It should be continued two, three, or four weeks, unless it fulfils the intention before that time. It would almost appear, that this remedy has been rejected by English practitioners in consequence of that devouring rage for POLYPHARMACY, which is at once the bane and disgrace of British medicine! When the day arrives (and arrive it must) that will award the remuneration for skill and attendance instead of drugs, then will the *external* remedies be more employed—and the nitro-muriatic solution again come into favour, Mr. A. cautions us against the employment of mercurials during the use of the solution—but recommends purgatives to carry off diseased secretions.

"We have frequently observed, after it has been employed for a few days, that the patient has complained much of heaviness or drowsiness. When this is the case, active purgation should be instituted, in addition to the use of the solution, which will soon bring away morbid and offensive stools, and remove this symptom of disorder." 629.

Change of air, or a sea voyage, will often materially assist the salutary operation of the acid bath.

III. *Nitrous Acid.* This medicine has been a good deal employed internally, as an alterative, in hepatic diseases. As much as six drachms of the diluted nitric acid, in a large quantity of barley or rice water, may be taken in 24 hours. In the course of a few days it induces a slight salivation; but its beneficial effects are often observed from smaller doses, and without the salivary excitation. This medicine requires a longer administration than mercury, in order to insure its full operation. There is no incompatibility in the use of this medicine with mercury. Indeed, it is the opinion of Sir James M'Grigor, as well as of our author, that the conjoined administration of both, is superior to that of either singly.

To attempt to impregnate the system with mercury, in the active forms of hepatitis—or in many cases of the chronic form, before the inflammatory action is, to a certain extent, subdued, leads, Mr. A. thinks, to want of success—nay, to positive injury. These objections do not apply to the nitrous acid, or to the nitro-muriatic bath; for, although these last are more serviceable after antiphlogistic measures have been employed, their premature use is not attended with any detriment.

IV. *Blisters, Setons, &c.* Blisters, of course, are not to be employed in the more acute forms till after depletion, on the same principle as in pneumonia, or any other internal inflammation. Afterwards they are salutary. In the more chronic and protracted cases, the drain of a seton or issue is more effectual. After a discharge has been established, Mr. A. recommends poultices to the part. We are a little surprised that Mr. A. does not mention the counter-irritation and purulent discharge produced by tartrite of antimony—an application extremely beneficial—and capable of being repeated from time to time without the constant inconvenience and pain of a seton. Tartar-emetic plaster is much superior to the ointment.

V. *Tepid and Vapour Baths.* These are serviceable in the course of the disease, whether in the active or chronic forms. In the *latter*, they should be followed by frictions, either with a coarse towel or flesh-brush, over the region of the liver, and, indeed, over the abdomen generally. The sulphur and chlorine baths are still more serviceable.

VI. *Eccoprotics.* "In the more chronic cases of hepatic disease, in addition to the external means already recommended, and particularly after local depletions have been resorted to whenever pain or uneasiness in the region of the liver manifested itself, a gentle aperient pill should be taken at bed-time, and saline laxatives through the day. The best pill which we can recommend for this purpose is that composed of the aloës and myrrh pill and blue pill, or the following:—℞. Hydr. submur. ℈j.; extract. colocynth. comp. ℈ij.; antim. tartar. gr. j.; pulv. ipecac.

gr. jv.; sapon. Castil. gr. x; ol. car. q. s. M. ft. pilul. xviij. Two of these will generally be found to operate sufficiently, and may be taken every night at bed-time, or every other night. In the majority of cases, however, one of them will prove sufficient, particularly when it is intended to continue the use of them for a considerable time, and when saline or other laxatives are also required through the day. Where the chronic disease of the liver is attended with enlargement, it will generally be found requisite to prescribe the above pills every night, the nitro-muriatic wash being employed externally night and morning; and a weak solution of the sulphates of soda, magnesia, and potash, either singly or combined, may be given in the morning, and, if necessary, again at mid-day, in order to keep up a gentle action in the large secreting viscera and bowels. If, however, a weak solution of these salts should occasion frequent and watery motions, with tenesmus, they may be changed for the solution of cream of tartar in tamarind water, or for the solution of the soda tartarizata, or the tartras potassæ. On many occasions, the factitious Cheltenham or Harrowgate salts may be given with advantage; and the Seidlitz powders may also be taken occasionally. Much benefit will generally accrue from changing, after a few days, the saline substances prescribed, particularly if the exhibition of the eccoprotic pill at bed-time, and the salts through the day, produce any degree of tenesmus. The cream of tartar solution may, however, be given and continued for a longer time, without any risk of inducing this effect. If tenesmus occur, an emollient enema will always afford relief, and the medicines may be intermitted for a day or two." 635.

From considerable experience in this class of complaints, we would suggest a modification of the above eccoprotic pill, which we have found to be an improvement. We would substitute the pilula hydrargyri for the calomel, and we would double the quantity of the tartrite of antimony. As there is often much irritation prevailing in the bowels, and in the system generally, three grains or four of extract of hyosciamus at bed-time, will be found a very useful adjuvant.

VII. *Bitters, Tonics, &c.* The continued operation of deobstruents—the defective nutrition—the impaired digestion—the irritative fever which more or less obtains—all these tend to produce great languor, debility, and irritability, in the hepatic invalid. On these accounts, it is very generally necessary to combine bitters or even tonics with the alterative or deobstruent remedies, in order to support the strength and improve the digestive and assimilative processes. The infusions of calumba, gentian, chamomile, with some tincture of the same, and soda, are the usual forms of bitter medicines—to which is to be added the taraxacum. We are very much surprised that this remedy does not enter into the catalogue which Mr. Annesley has laid before his readers. As his experience has been almost entirely confined to India, where the taraxacum is little used, Mr. A. probably is practically unacquainted with its effects. But the more it is employed, the more certain proofs will it afford of its utility. The aperient and diuretic qualities of this root are unquestionable. The following will be found a very convenient, and not an unpleasant formula.

℞. Decocti taraxaci . . . . . . . . . . . . . . ℥iv.

Carb. sodæ . . . . . . . . . . . . . . . . ʒj.

Extr. taraxac . . . . . . . . . . . . . . . . ʒij.

Tinct. gent. c. . . . . . . . . . . . . . . . ʒij.

Misce fiat mistura, capiat coch. ij. vel. iij. mag. bis die.

Although tonics are precarious medicines, and can rarely be given while any inflammatory action is going on in any of the chylopoietic organs, yet a period sooner or later arrives when they become necessary, and are then highly beneficial. They should be preceded by bitters, and their doses should be very small at the beginning. It is by giving too much for a dose, or too many doses in the day—or by exhibiting the tonic at an improper time, that mischief is done. Thus many people will derive advantage from half a grain of sulphate of quinine in a drachm of compound tincture of gentian taken *directly after dinner*, whose stomachs would be irritated or too much excited by the same dose, an hour before dinner, or two or three times in the day. But the unfortunate mode of medical remuneration in this country, destroys very often the efficacy of a medicine by the manner in which it is obliged to be administered.

Mr. Annesley has evidently a *hobby* in the shape of purgation. In India, where the biliary apparatus is goaded to inordinate and morbid secretion, by the heat of the climate, and the stimulation of diet and drink, purgation is necessary, even to the amount prescribed by our author. But in this country, where the liver is generally torpid, and the stomach and bowels irritable, the drastic purgation recommended by Mr. Annesley, would be found a very inconvenient, if not a detrimental practice. The diet and regimen of an hepatic patient require peculiar attention—more than we can speak of in this place. Mr. Annesley has touched very lightly on this important subject—and he seems to have entirely over-looked the salutary effects of TRAVELLING in diseases and disorders of the biliary and chylopoietic organs. This item would often do more for the Indian invalid than the "purging discipline" (to use a favourite expression of Mr. Annesley) which is so strenuously recommended in the great work under review. But we must now bring this article to a close. We shall take up another subject, in the course of our next number, and pursue our analyses till we exhibit a complete view of these two costly and highly valuable volumes.

